# Combined Boyden-Flow Cytometry Assay Improves Quantification and Provides Phenotypification of Leukocyte Chemotaxis

**DOI:** 10.1371/journal.pone.0028771

**Published:** 2011-12-09

**Authors:** Nardhy Gomez-Lopez, Felipe Vadillo-Ortega, Guadalupe Estrada-Gutierrez

**Affiliations:** 1 Research Direction, Instituto Nacional de Perinatologia Isidro Espinosa de los Reyes, Mexico City, Mexico; 2 Department of Obstetrics and Gynecology, University of Alberta, Edmonton, Alberta, Canada; 3 Discipline of Obstetrics and Gynaecology, The University of Adelaide, Adelaide, South Australia, Australia; 4 Biochemistry Department, Faculty of Medicine, Universidad Nacional Autonoma de Mexico, Mexico City, Mexico; 5 Department of Infectology, Instituto Nacional de Perinatologia Isidro Espinosa de los Reyes, Mexico City, Mexico; University of Regensburg, Germany

## Abstract

Chemotaxis has been studied by classical methods that measure chemotactic and random motility responses *in vitro*, but these methods do not evaluate the total number and phenotype of migrating leukocytes simultaneously. Our objective was to develop and validate a novel assay, combined Boyden-flow cytometry chemotaxis assay (CBFCA), for simultaneous quantification and phenotypification of migrating leukocytes. CBFCA exhibited several important advantages in comparison to the classic Boyden chemotaxis assay (CBCA): 1) improved precision (intra-assay coefficients of variation (CVs): CBFCA-4.7 and 4.8% vs. CBCA-30.1 and 17.3%; inter-observer CVs: CBFCA-3.6% vs. CBCA 30.1%); 2) increased recovery of cells, which increased assay to provide increased sensitivity; 3) high specificity for determining the phenotype of migrating/attracted leukocytes; and 4) reduced performance time (CBFCA 120 min vs. CBCA 265 min). Other advantages of CBFCA are: 5) robustness, 6) linearity, 7) eliminated requirement for albumin and, importantly, 8) enabled recovery of migrating leukocytes for subsequent studies. This latter feature is of great benefit in the study of migrating leukocyte subsets. We conclude that the CBFCA is a novel and improved technique for experiments focused on understanding leukocyte trafficking during the inflammatory response.

## Introduction

Leukocyte infiltration is a crucial event of inflammation. This process is regulated by several molecules, including chemoattractants and cellular adhesion molecules [1]. Chemoattraction is termed chemotaxis when the ligand gradient is soluble. Chemotaxis is described as an ensemble of cell activities that are precisely orchestrated in space and time, consisting of highly dynamic cell shape rearrangements accompanied by rapid adhesion–de-adhesion cycling and directional movement [2].

Different models for investigating leukocyte chemotaxis have been developed to measure chemotactic and random motility responses *in vitro*. These assays provide information on the locomotion of leukocytes, which is not only of physiological interest, but also important in furthering our understanding of the inflammatory response. Although these assays do not necessarily mimic *in vivo* conditions, they are useful tools for studying the cellular functions that occur in living tissues [3], [4], [5], [6].

The most common methods used to evaluate chemotaxis are those in which leukocytes migrate through a micropore membrane [7], of which the Boyden model is an early example [3]. Although this technique provides an estimation of the relative leukocyte chemotactic activity of soluble substances, it is suggested that it gives partial and sometimes misleading information, and should be supplemented with other methods including visual assays [8]. Consequently, the Boyden chamber technique has been modified over the years [9], [10], [11]; however, none of these modifications allow the quantification of total migrating leukocytes concurrently with the determination of their phenotype.

Phenotypification of migrating leukocytes is not required for chemotaxis studies that utilize purified leukocyte subsets, such as neutrophils [12] and monocytes [13]. However, in many experimental paradigms, the simultaneous evaluation of distinct leukocyte subsets is crucial [12], [14], [15], and this typically requires separate assays. To associate specific inflammatory responses with distinct leukocyte subsets, investigators require an improved method for studying the density and phenotype of diverse leukocyte subsets simultaneously. In the present study we describe and validate an improved CBFCA, which allows the accurate quantification and phenotypification of different migrating leukocyte subsets in a single assay. This CBFCA allows the accurate quantification and recovery of the migrating leukocytes for subsequent phenotypification and characterization in a single assay, which facilitates understanding of leukocyte trafficking during the inflammatory response.

## Materials and Methods

### Ethics statement

This study was approved by the institutional review board of the Instituto Nacional de Perinatologia Isidro Espinosa de los Reyes in Mexico City (Register 212250-02121). Written and informed consent was obtained from each subject prior to inclusion in the study.

### Isolation of leukocytes

Peripheral blood samples from healthy female donors (age 30–40 years) were collected into heparinized tubes. Total leukocytes were isolated using Polymorphprep™ (Axis-Shield, MA, USA) density gradient according to the manufactureŕs instructions. Following centrifugation, both bands of leukocytes (mono-and polymorphonuclear) were recovered and mixed. Total leukocytes were washed twice in calcium and magnesium-free 1X phosphate buffered saline (PBS) at 4°C and resuspended in Dulbeccós modified Eagle's medium (DMEM) (GIBCO Invitrogen, CA, USA). Leukocyte viability was assessed by trypan blue exclusion and the leukocyte count was determined using a Beckman Coulter AcT 5 Diff Hematology Analyzer (Beckman Coulter, CA, USA). Viability was greater than 97%, with negligible contamination by red blood cells. Leukocyte suspensions were stored at 37°C and used within an hour of isolation.

### CBCA

**Figure 1:**
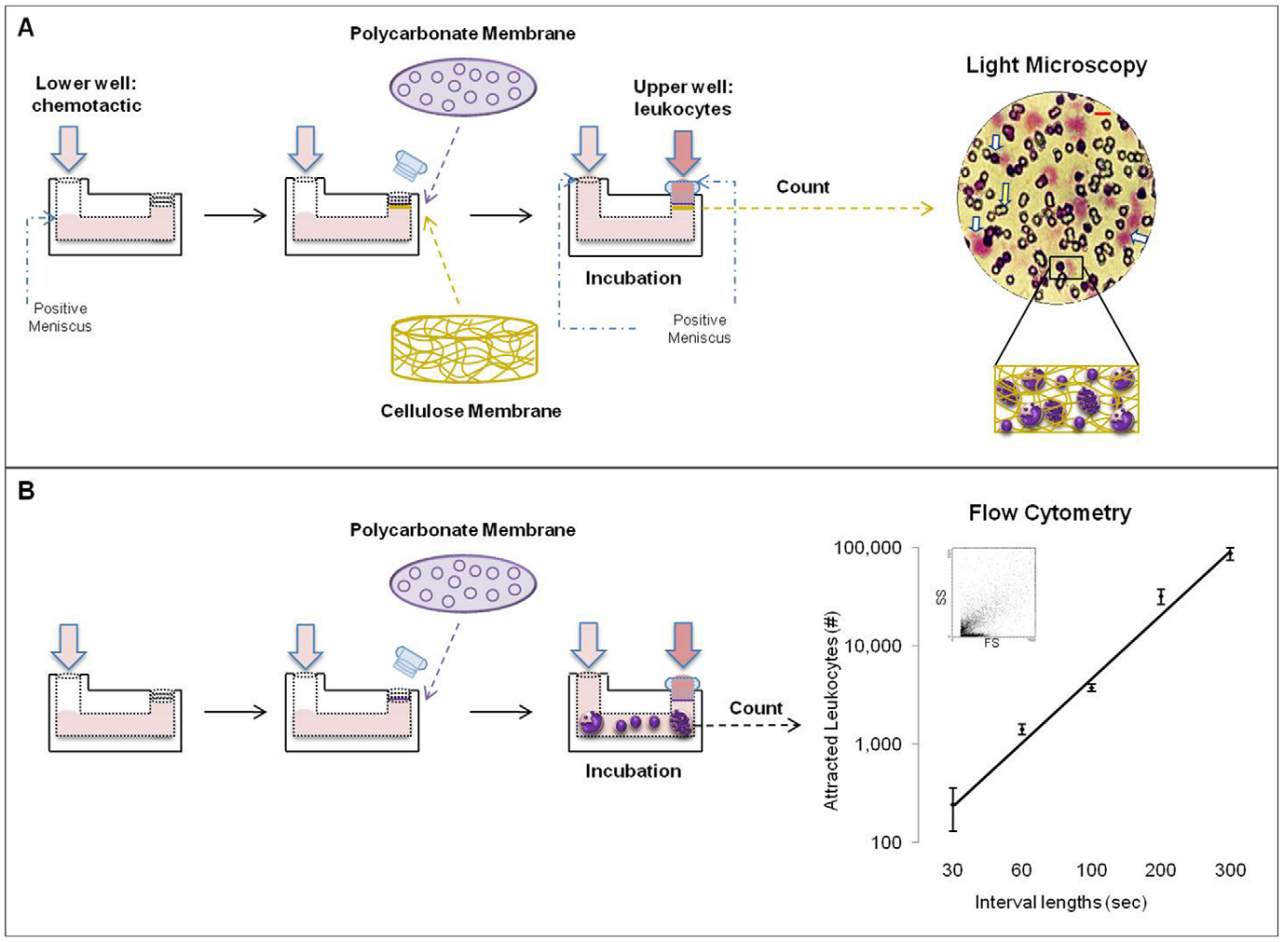
CBCA & CBFCA. **A)** CBCA includes a permeable polycarbonate membrane and a second cell impermeable cellulose membrane directly beneath to trap attracted cells. These membranes separate the upper well containing leukocytes and the lower well containing the chemotactic. Attracted leukocytes are counted in the cellulose membrane under light microscopy in different focal planes (magnification). White arrows represent the leukocytes trapped and agglutinated into the cellulose membrane, and the yellow arrow represents its pores. Light microscopy: magnification x400; Red bar 10 µm. **B)** CBFCA includes a single polycarbonate membrane between the wells. Attracted leukocytes in the lower well are counted by flow cytometry and their number increases lineally with the length of interval. The leukocyte phenotype can also be determined in this step.

The classic chemotaxis assay was performed according to the method described by Snyderman, *et al.* [16] using modified Boyden chambers (BY312; Neuro Probe, MD, USA) and a double membrane modification [17] (Fig. 1A). The lower well of the chamber was filled creating a slight positive meniscus with 1.2 ml of chemotactic (either medium as control or 10^−6^ M N-Formil-Met-Leu-Phe (fMLP) (Sigma-Aldrich, MO, USA) diluted in DMEM). This concentration of fMLP has been shown to be optimal for inducing migration of leukocytes [18]. Two membranes were used together to separate the chamber wells: a 5 µm pore size polycarbonate membrane, 22 µm in thickness (TMTP01300, Millipore Corporation, MA, USA) to select the cells, and a 8 µm pore size cellulose membrane, 160 µm in thickness (SCWP01300, Millipore Corporation) directly above the lower well to trap the cells. Then, the thumb nut was placed and tightened until finger-tight. The upper and lower wells were then filled simultaneously creating a slight positive meniscus, the lower with 500 µL of chemotactic and the upper with 500 µL of leukocyte suspension containing 5×10^5^ leukocytes. Boyden chambers were incubated in a humidified atmosphere at 37°C for 90 min [13], and the horizontal position was assured using a bubble level device. Following incubation, the cellulose membrane was removed, washed in 1X PBS, fixed with methanol, stained with Harris hematoxilin-eosin, and mounted on glass slides using Entellan® (Electron Microscopy Sciences, PA, USA) with the upper surface facing up. Migrating total leukocytes were counted by light microscopy at 400 X using an oil-immersion objective. Two perpendicular diameters were counted on each membrane (>100 fields per membrane) [13], [19] and the result was scored as the number of attracted leukocytes per membrane. Leukocytes attracted by the control were subtracted in all cases.

### CBFCA

The combined chemotaxis assay was performed under the same conditions described for the classic method. However, only the polycarbonate membrane was used (Fig. 1B). Following incubation, the leukocyte suspension in the lower well was removed and centrifuged at 500×*g* for 5 min at room temperature. The pellet containing attracted leukocytes was fixed with 500 µL of Optilyse B (Beckman Coulter), washed and resuspended in 500 µL of 1X PBS, and leukocytes were counted using a flow cytometer (FC500; Beckman Coulter). Leukocyte counting by flow cytometry (using a medium flow rate) was evaluated at six different counting length intervals (30, 60, 100, 200, 300 sec) using 10^−6^ M fMLP and a standard leukocyte sample. As for CBCA, leukocytes attracted by the control were subtracted in all cases.

### Validation of CBFCA

For validation of the performance of this proposed assay, we have considered the classical parameters of precision, accuracy, sensitivity, and specificity.

#### Precision

The intra-assay variation was determined by performing two sets of assays, using 10^−6^ M fMLP as chemotactic and leukocytes from the same person. Set 1 included 12 assays and set 2 included 15 assays. Both sets were performed using both methods CBCA or CBFCA, and using two different leukocyte samples. The inter-observer variation was determined by using 6 different expert observers/operators, where 3 counted by microscopy (CBCA) and 3 counted by flow cytometry for 100 sec (CBFCA). The coefficients of variation were calculated by dividing the standard deviation of the estimates by their mean.

#### Accuracy and sensitivity

The classical definitions of these validation terms are difficult to apply to this assay because of the lack of an accepted “gold standard”. To address these concepts, we performed 10 independent assays in triplicate (each independent assay included 1 sample of leukocytes): 5 assays using 10^−6^ M fMLP as standard chemotactic, and 5 assays using plasma obtained from healthy donors as biological chemotactic [20]. Assays were performed using CBCA or CBFCA. We calculated the recovery of the cells after the assay and then considered the accuracy and sensitivity of the counting technique to provide valid measurements.

#### Specificity

Again, the concept of specificity is difficult to apply to this assay. To demonstrate the ability of the CBFCA to distinguish among phenotypes in a mixed population of leukocytes, we used flow cytometry to assess the leukocyte phenotype before and after performing CBFCA, using 10 µL of each of the following fluorescent monoclonal antibodies (Beckman Coulter): CD45-FITC (PN IM2643) for total leukocytes, CD3-PC7 (6607100) for T lymphocytes, CD19-PC5 (PN IM2643) for B lymphocytes, CD14-ECD (PN IM2707U) for monocytes, and CD56-PE (PN IM2073) for NK cells. Leukocyte subsets were analyzed within the CD45+ region considering 10,000 events. Granulocytes were identified as CD45^+^CD3^-^CD19^-^CD14^-^CD56^-^ cells. Specificity of CBFCA was observed by comparing the leukocyte subset proportions before and after assay. Although we only analyzed general leukocyte subsets, each laboratory will chose specific antibodies according to its needs.

### Robustness

We evaluated the leukocyte chemotaxis of a biological chemotactic at different concentrations in time course experiments. CBFCAs were performed using both undiluted and diluted plasma (1∶50 and 1∶100). Since CBFCA also determines the phenotype of attracted leukocytes and each leukocyte subset has different responsiveness [21], we established the robustness of the incubation time for each leukocyte subset in CBFCA. Assays at different incubation times (60, 90 and 120 min) were performed using plasma as biological chemotactic.

### Linearity

CBFCAs were performed using two purified leukocyte subsets; neutrophils (5×10^5^, 5×10^4^, 5×10^3^ and 5×10^2^) and monocytes (5×10^3^ and 5×10^2^), with 10^−3^ or 10^−6^ M fMLP as chemotactics. Neutrophils were purified with Polymorphprep™ solution (Axis shield), and monocytes were positively separated by magnetic cell sorting, using the Monocyte Isolation Kit (Miltenyi Biotec, CA, USA), according to the manufacturer's instructions.

### Use of Albumin

It has been reported that an exogenous protein source is required to obtain reproducible results when chemotaxis is evaluated using cellulose membranes [7]. However, it is unknown if addition of albumin effects the quantification of the CBFCA. This was evaluated by performing CBFCAs using medium and 10^−6^ M fMLP, in the absence or presence of two previously reported concentrations of human albumin (HA) (wt/vol): 0.1% [22], [23], [24] and 2% [25], [26].

### Statistical analyses

The data were examined initially by the Shapiro-Wilk test for normal distribution. Since they were normally distributed, T tests, ANOVA and post-hoc tests were used according to the homogeneity of variances. Statistical analyses were performed using SPSS version 18.0. A P value of<0.05 was considered statistically significant.

## Results and Discussion

### Comparison between CBCA and CBFCA

Several variations of the original Boyden technique have been reported since it was described [8], [27]. Snyderman introduced the double membrane assay (CBCA), which is widely used [9], [16], [17], [19]. Migrating leukocytes pass through the polycarbonate membrane by a process similar to diapedesis [1] and become trapped in the cellulose membrane. These trapped leukocytes were counted by light microscopy. Although this technique is commonly used to investigate chemotaxis *in vitro* [3], [27], [28], the assessment of the number of attracted leukocytes is subjective and time-consuming. Leukocyte counting by CBCA involved approximately 2 hrs per membrane/assay. Variation arises due to trapping and clustering of the leukocytes in different focal planes of the cellulose membrane (Fig. 1A). In CBFCA, the cellulose membrane was eliminated, enabling recovery of leukocytes in a fluid medium after passing through the polycarbonate membrane. Interval lengths of 30, 60, 100, 200, 300 sec were used for leukocyte counting by flow cytometry (Fig. 1B). Here, we demonstrated that the leukocyte count increased logarithmically with the interval length. We recommend that each laboratory chooses this parameter according its experimental design. We selected 300 sec because it maximizes the number of counted cells and remains in the linear portion of the relationship between time and leukocyte count.

### Validation of CBFCA

#### Precision

**Figure 2:**
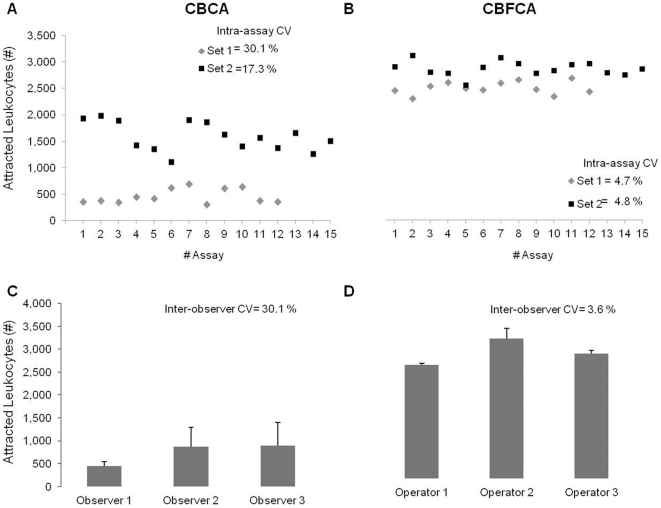
Precision: Intra-assay and inter-observer variation. **A&B)** Two sets of assays were performed by CBCA or CBFCA, set 1 includes 12 assays (gray diamonds) and set 2 includes 15 assays (black squares). Intra-assay coefficient of variation (CV) in CBCA was higher than in CBFCA in both sets (30.1 and 17.3% vs 4.7 and 4.8%). **D&C)** Three of these assays were counted by three different observers/operators using microscopy or flow cytometry. The inter-observer CV in CBCA was higher than in CBFCA (30.1 vs 3.6%). Data are shown as means±SD of three independent assays counted in triplicate.

To determine the precision of both assays, we calculated the intra-assay variation and a homologous of an inter-assay, the inter-observer variation. In both sets of assays, the intra-assay CV was higher in CBCA (30.1 and 17.3%) than in CBFCA (4.7 and 4.8%) (Fig. 2A and B). In addition, the inter-observer CV was higher in CBCA (30.1%) than in CBFCA (3.6%) (Fig. 2C and D). The high inter-observer CV in CBCA was attributed to the assay rather than the observers, since the three observers had high expertise and experience in microscopy. These data demonstrate that CBFCA exhibits improved precision over CBCA.

#### Accuracy and sensitivity

Using fMLP as the standard chemical chemoattractant and human plasma [20] as a biological chemoattractant, we determined the accuracy of both methods. While CBFCA determined 90% of the leukocyte chemotaxis of plasma, CBCA only determined 29%. In addition, the counting range in the CBCA method was 200 – 2500 cells per membrane (up to 0.5 % of 5×10^5^ placed cells) and in the CBFCA method was markedly expanded to 100–100,000 cells per tube (up to 20% of 5×10^5^ placed cells). Furthermore, when we used purified leukocyte subsets, CBFCA increased its counting range even further to 100 – 250,000 cells (up to 50% of 5×10^5^ placed cells). These data strongly suggest that the sensitivity and range of measurability of CBFCA is superior to CBCA. It is apparent that the number of cells applied to the assay and the interval length used for counting by flow cytometry will have a significant impact on the ability of the method to produce valid measurements. We recommend that each laboratory consider these parameters before using this assay and individualize the parameters according to their experimental conditions and desired end-points.

#### Specificity

**Figure 3:**
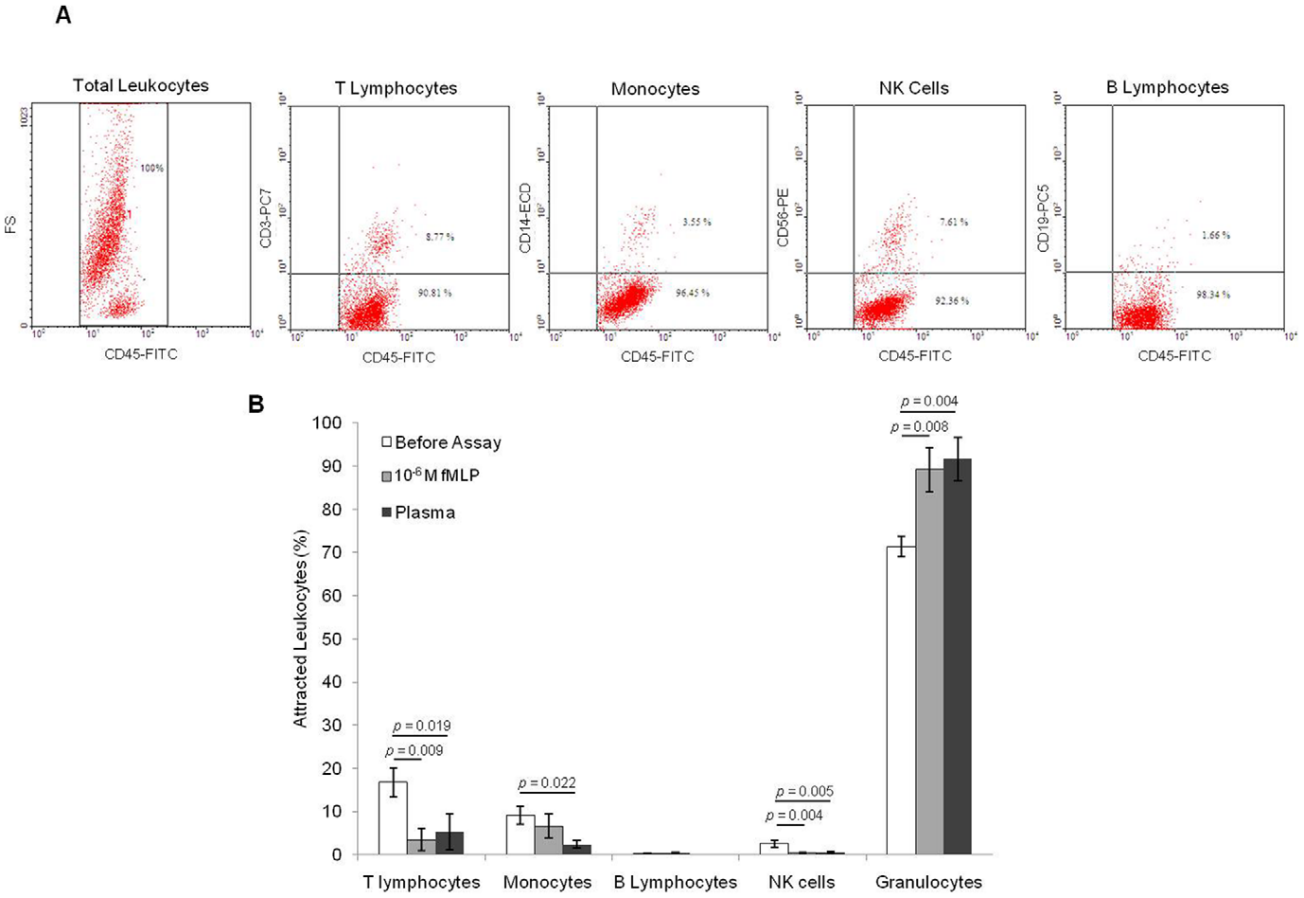
Specificity of CBFCA. CBFCA involves the determination of the leukocyte phenotype using conjugated specific antibodies and flow cytometry. **A)** Representative dot-plots showing the dual parameters used for the flow cytometric analysis: CD45+ for total leukocytes, CD45+CD3+ for T-lymphocytes, CD45+CD14+ for monocytes, CD45+CD56+ for NK cells and CD45+CD19+ for B lymphocytes. **B)** Specific leukocyte chemotactic activity of 10^−6^ M fMLP and plasma. Leukocyte subset proportions were significantly different before and after the assay. Data shown are means±SD of five independent assays in triplicate. Significance was determined by ANOVA and Games-Howell test.

It was not possible to determine the specificity of CBCA, since in this step the attracted leukocytes were trapped into the cellulose membrane and the phenotype determination was subjective. The specificity of CBFCA was evaluated by determination of the phenotype of attracted leukocytes using specific conjugated antibodies and flow cytometry (Fig. 3A). Our data showed that 10^−6^ M fMLP and plasma exhibited specific leukocyte chemotactic activity, since the proportion of leukocyte subsets differed before and after assay: T lymphocytes (*p = *0.009 and 0.019), monocytes (*p = *ns and 0.022), NK cells (*p = *0.004 and 0.005), and granulocytes (*p = *0.008 and 0.004). Since fMLP and plasma are granulocyte/neutrophil chemotactics [20], [29], we did not see significant differences between them (Fig. 3 B). These data demonstrates that CBFCA permits the determination of the specific leukocyte chemotactic activity of commercial and biological chemotactics. Boyden chamber assays and flow cytometry have been conjugated before for studying leukocyte chemotaxis [30]; however, none of these studies used the flow cytometric analysis for the quantification and phenotypification of the attracted leukocytes simultaneously. The specificity of CBFCA for determining the phenotype of migrating leukocytes depends of antibodies and cytometer used in each laboratory. All the antibodies used were 97–100% specific.

### Other advantages of CBFCA

#### Robustness

**Figure 4:**
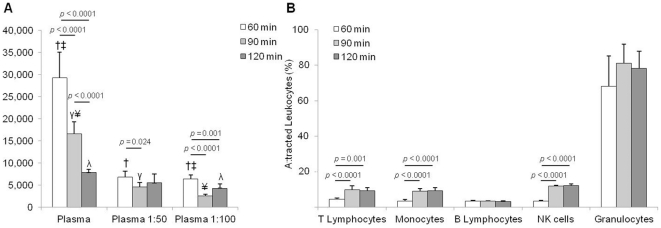
A) Robustness of CBFCA. To investigate the robustness of CBFCA the leukocyte chemotactic activity of undiluted and diluted plasma was determined in time course experiments. CBFCA was able to determine the biological chemotactic activity of diluted and undiluted plasma at 60, 90 and 120 min. **B)** Robustness of the incubation time in CBFCA. CBFCAs were performed at different incubation times (60, 90 and 120 min) using total leukocytes and plasma. All the leukocyte subsets reached the top of their migration within 90 min and their proportions did not change at 120 min of incubation time. Granulocytes and B-lymphocytes completed their migration within the first 60 min. Data shown are means±SD of five independent assays in triplicate. Means with same symbol are significantly different. Significance was determined by ANOVA and Games-Howell tests (*p*≤†‡γ¥ 0.0001, λ 0.005).

We evaluated the robustness of CBFCA by quantifying the leukocyte chemotaxis of diluted and undiluted plasma at different incubation times. Our results indicate that undiluted plasma induced more leukocyte chemotactic activity than diluted plasma at 60, 90 or 120 min of incubation (*p<*0.005) (Fig. 4 A). These results indicate that CBFCA is robust enough to quantify the attracted leukocytes in a wide range of biological concentrations of chemotactics. This represents an advantage when small (and diluted) volumes of biological samples are available for chemotaxis studies.

The choice of incubation time in the chemotaxis assays is crucial for the evaluation of the number of attracted leukocytes [31]. We performed time course experiments using undiluted and diluted plasma. With this set of experiments, it was demonstrated that the migration rate was higher at 60 than at 90 min of incubation, regardless of using undiluted or diluted plasma (*p<*0.024). In addition, the migration rate is higher at 90 than at 120 min of incubation when undiluted plasma is used (*p<*0.0001) (Fig. 4 A). These results indicate that the incubation time for the CBFCA depends of the chemoattractant concentration. Moreover, the fact that each leukocyte subset has different responsiveness to chemotaxis [7], [21] should be considered. Since we mostly use CBFCA with mixed leukocyte subsets, we performed assays at three different incubation times, using total leukocytes and undiluted plasma. We demonstrated that CFBCA had a robust incubation time of 30 min, which comprised between the 90 and 120 min of incubation time. Thus, we observed that all the leukocyte subsets reached the top of their migration within 90 min and their proportions did not change at 120 min of incubation time (Fig 4 B). This represents an advantage of this assay when the incubation time needs to be modified according the experimental design. We also demonstrated that granulocytes and B-lymphocytes completed their migration within the first 60 min (Fig 4 B). This represents an advantage of quick incubation time when purified granulocyte or B lymphocyte subsets are tested in CBFCA. 1∶50 and 1∶100 plasma dilutions caused the same changes in proportions of leukocyte subsets (data not shown).

#### Linearity

**Figure 5:**
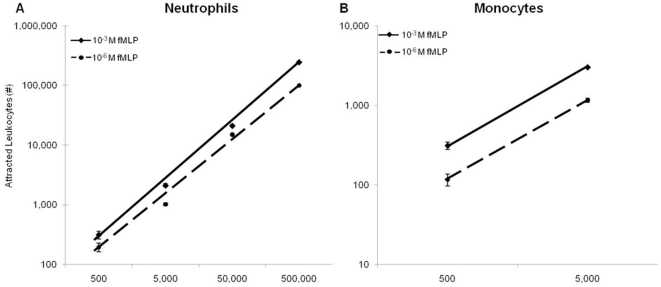
Linearity of CBFCA. CBFCAs were performed using 10^−3^ and 10^−6^ M fMLP as chemotactic and purified neutrophils (**A**) and monocytes (**B**) at different concentrations. The number of attracted neutrophils and monocytes increased lineally with the starting number of cells. Significantly more leukocytes were attracted by 10^−3^ M fMLP than by 10^−6^ M fMLP in all the cases. Data shown are means±SD of four independent assays in triplicate. Significance was determined by T test.

We demonstrated the linearity of CBFCA, using purified neutrophils and monocytes and two concentrations of fMLP (10^−6^ and 10^−3^ M). Our data showed that the number of attracted neutrophils and monocytes increased lineally with the starting number of cells. This is an important consideration of the quantitative validity of the end-point measurements. As expected, 10^−3^ M fMLP attracted more leukocytes than 10^−6^ M fMLP in all the cases (*p<*0.0001). This is further evidence of the *sensitivity* of CBFCA, since we observed differences of leukocyte chemotactic activity using the minimal starting number for attraction (500 cells). In addition, although the reported optimal concentration of fMLP for migration is 10^−6^ M, we demonstrated that CBFCA permitted testing of more concentrated chemotactics (10^−3^ M fMLP). This represents other evidence of *robustness* in CBFCA (Fig. 5). We recommend the optimization of leukocyte and chemotactic concentrations before using this assay.

#### Performance Time

The time required for sample preparation and incubations are similar in CBCA and CBFCA. However, the time required for staining and cell counting in the latter is reduced by 2–3 hours. This indicates that not only does the CBCFA method provide important additional information regarding leukocyte phenotype; it can be performed in approximately half the time required for the CBCA.

#### CBFCA does not require albumin

**Figure 6:**
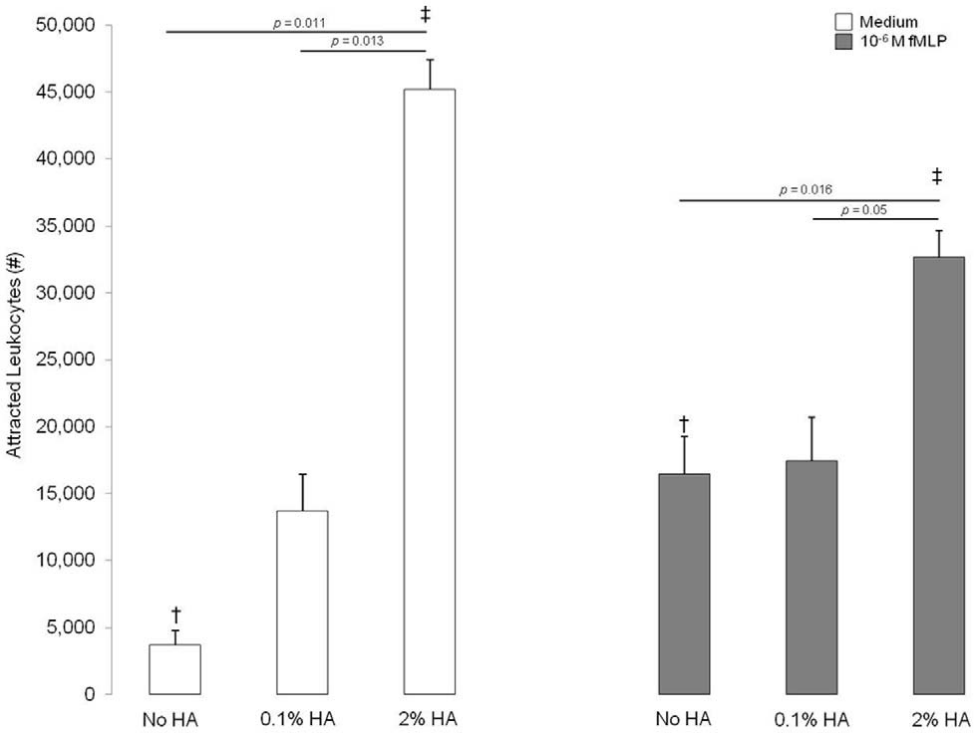
Effect of HA on leukocyte chemotaxis. CBFCAs were performed using medium or 10^−6^ M fMLP, in the absence or presence of 0.1% and 2% HA. The addition of 2% HA to the medium and 10^−6^ M fMLP attracted more leukocytes; however, it also masked the chemotactic activity of 10^−6^ M fMLP. Data are shown as means±SD of five independent assays in triplicate. Means with same symbol are significantly different. Significance was determined by ANOVA, Games-Howell tests and T test (*p = *†‡ 0.027).

Many researchers have utilized human albumin (HA) to facilitate the chemotaxis process [8], [22], [23], [24], [25], [26]. However, these previous investigations did not consider that HA could confound or mask the chemotactic activity of specific chemotactics. Figure 6 demonstrates the interaction between HA and fMLP on leukocyte chemotaxis evaluated by CBFCA. Albumin itself has significant chemotactic activity (Figure 6, white bars). However, in the presence of 2% HA, the chemoattractant activity of the standard chemical chemotactic agent fMLP was significantly underestimated or “masked” (Figure 6, gray bars). Thus, all our assays were performed without HA to enable a clear evaluation of the biological chemotactic activity. Although the addition of HA has been recommended in assays with monocytes to maintain their viability [32], we suggest avoiding its use for the proposed CBFCA.

### Disadvantages of CBFCA

As mentioned CBFCA is a modern assay and therefore there is also a disadvantage; it is more expensive than CBCA since it involves the use of antibodies and flow cytometry. Flow cytometry performance also requires a trained operator and reagents. Overall, we consider that the advantages offered by using CBFCA compensated for the expensiveness of this assay.

In summary, we have demonstrated that CBFCA is a precise, specific, sensitive, and robust tool for the *in vitro* quantification of leukocyte chemotactic activity. Our method enables rapid quantification and simultaneous phenotypification of migrating leukocytes, and it is adaptable to biological samples and commercial chemotactics. Importantly, this is the first method that permits the recovery of migrating leukocytes for subsequent analysis and characterization. Knowing the pattern and phenotype of migrating leukocytes, novel therapeutic approaches can be developed to target specific leukocyte subsets implicated in immunopathological responses. In summary, the information attainable by the routine use of this combined assay will further our understanding of both the chemotaxis process and the inflammatory response.
